# 1-[(*Z*)-2-Cyano-2-(2-pyrid­yl)vin­yl]­ferrocene

**DOI:** 10.1107/S1600536808021843

**Published:** 2008-07-19

**Authors:** Xue-Qun Fu, Wei Wang

**Affiliations:** a Ordered Matter Science Research Center, Southeast University, Nanjing 210096, People’s Republic of China

## Abstract

In the title compound, [Fe(C_5_H_5_)(C_13_H_9_N_2_)], the dihedral angle between the substituted cyclo­penta­dienyl plane and the plane of the pyridine ring is 8.43 (14)°. The double bond adopts a *Z* configuration. In the crystal structure, weak C—H⋯N inter­actions link the molecules into a zigzag chain. A weak intramolecular C—H⋯N hydrogen bond is also present.

## Related literature

For the chemistry of ferrocene, see: Chen *et al.* (2006 ▶). For representative ferrocene derivatives, see: Jiao *et al.* (2003 ▶); Mancheno *et al.* (2004 ▶). For similar compounds, see: Boyd & Paauwe (2006 ▶); Shao *et al.* (2005 ▶).
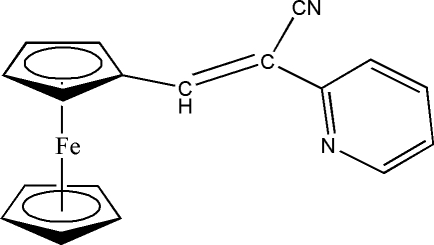


## Experimental

### 

#### Crystal data

[Fe(C_5_H_5_)(C_13_H_9_N_2_)]
*M* *_r_* = 314.16
Monoclinic, 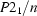

*a* = 11.105 (2) Å
*b* = 10.716 (2) Å
*c* = 12.675 (3) Å
β = 106.95 (3)°
*V* = 1442.9 (5) Å^3^
*Z* = 4
Mo *K*α radiation
μ = 1.04 mm^−1^
*T* = 293 (2) K
0.30 × 0.20 × 0.10 mm

#### Data collection

Rigaku Mercury2 diffractometer
Absorption correction: multi-scan (*CrystalClear*; Rigaku, 2005 ▶) *T*_min_ = 0.725, *T*_max_ = 0.900
14799 measured reflections
3311 independent reflections
2564 reflections with *I* > 2σ(*I*)
*R*_int_ = 0.045

#### Refinement

*R*[*F* ^2^ > 2σ(*F* ^2^)] = 0.041
*wR*(*F* ^2^) = 0.094
*S* = 1.07
3311 reflections
190 parameters
H-atom parameters constrained
Δρ_max_ = 0.24 e Å^−3^
Δρ_min_ = −0.24 e Å^−3^

### 

Data collection: *CrystalClear* (Rigaku, 2005 ▶); cell refinement: *CrystalClear*; data reduction: *CrystalClear*; program(s) used to solve structure: *SHELXS97* (Sheldrick, 2008 ▶); program(s) used to refine structure: *SHELXL97* (Sheldrick, 2008 ▶); molecular graphics: *SHELXTL* (Sheldrick, 2008 ▶); software used to prepare material for publication: *SHELXTL*.

## Supplementary Material

Crystal structure: contains datablocks I, global. DOI: 10.1107/S1600536808021843/bq2088sup1.cif

Structure factors: contains datablocks I. DOI: 10.1107/S1600536808021843/bq2088Isup2.hkl

Additional supplementary materials:  crystallographic information; 3D view; checkCIF report

## Figures and Tables

**Table 1:** Hydrogen-bond geometry (Å, °)

*D*—H⋯*A*	*D*—H	H⋯*A*	*D*⋯*A*	*D*—H⋯*A*
C16—H16*A*⋯N2	0.93	2.41	2.804 (3)	105
C4—H4*A*⋯N1^i^	0.98	2.62	3.538 (4)	156

Symmetry code: (i) 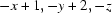.

**Fig. 1.**
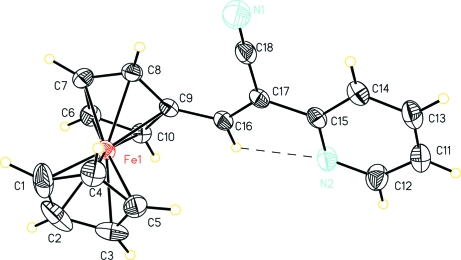
The molecular structure of the title compound, with displacement ellipsoids drawn at the 30% probability level. Hydrojen bond are shown as dashed lines

**Fig. 2.**
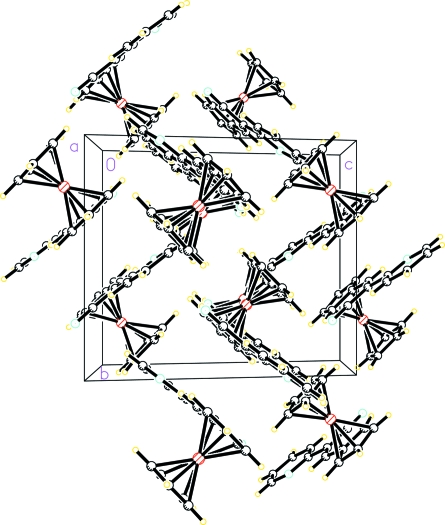
The packing diagram of the title compound, viewed along the a axis.

## supplementary crystallographic information

### Comment

The molecular structure of the title compound is shown in Fig. 1. The Fe···*Cg*1 and Fe···*Cg*2 distances are 1.6548 (16)Å and 1.6445 (12)Å respectively and the *Cg*1··· Fe··· *Cg*2 angle is 179.55 (8)°, where *Cg*1 and *Cg*2 are the centroids of the unsubstituted and substituted cyclopentadienyl rings. The double bond (C16═C17) exhibits a *cis* configuration and the pyridine plane makes an angle of 8.43 (14)Å with the substituted cyclopentadienyl ring. The planar cyclopentadienyl rings of the ferrocenyl unit are nearly parallel to each other [the interplanar angle is 1.33 (17)°]. The crystal structure is stabilized by weak intramolecular C—H···N interactions. Fig 2 shows that the molecules assemble as zigzag chains in the crystal structure along the *a* axis, formed by weak intermolecular C—H···N hydrogen bonds (Table 1).

### Experimental

1 ml pyrrolidine was added to the mixture of formylferrocene (2.15 g, 0.01 mol) and 2-pyridineacetonitrile (1.18 g, 0.01 mol) in dichloromethane (100 ml). The mixture was stirred at room temperature for 5 h. After removing the solvent under reduced pressure, the residue was collected and dried in a vacuum desiccator. This crude product was purified by chromatography on silica gel, with petroleum ether and ethyl as eluant. Brownish red single crystals suitable for X-ray analysis were obtained by slow evaporation of ether at room temperature after several hours.

### Refinement

Positional parameters of all the H atoms were calculated geometrically and were allowed to ride on the C atoms to which they are bonded, with *U*_iso_(H) = 1.2*U*_eq_(C).

### Crystal data

**Table:**

[Fe(C_5_H_5_)(C_13_H_9_N_2_)]	*F*_000_ = 648
*M**_r_* = 314.16	*D*_x_ = 1.446 Mg m^−^^3^
Monoclinic, *P*2_1_/*n*	Mo *K*α radiation λ = 0.71073 Å
Hall symbol: -P 2yn	Cell parameters from 12734 reflections
*a* = 11.105 (2) Å	θ = 6.7–55.3º
*b* = 10.716 (2) Å	µ = 1.04 mm^−^^1^
*c* = 12.675 (3) Å	*T* = 293 (2) K
β = 106.95 (3)º	Prism, red brown
*V* = 1442.9 (5) Å^3^	0.30 × 0.20 × 0.10 mm
*Z* = 4	

### Data collection

**Table:**

Rigaku Mercury2 (2x2 bin mode) diffractometer	3311 independent reflections
Radiation source: fine-focus sealed tube	2564 reflections with *I* > 2σ(*I*)
Monochromator: graphite	*R*_int_ = 0.045
Detector resolution: 13.6612 pixels mm^-1^	θ_max_ = 27.5º
*T* = 293(2) K	θ_min_ = 3.4º
CCD_Profile_fitting scans	*h* = −14→14
Absorption correction: Multi-scan(CrystalClear; Rigaku, 2005)	*k* = −13→13
*T*_min_ = 0.725, *T*_max_ = 0.900	*l* = −16→16
14799 measured reflections	

### Refinement

**Table:**

Refinement on *F*^2^	Secondary atom site location: difference Fourier map
Least-squares matrix: full	Hydrogen site location: inferred from neighbouring sites
*R*[*F*^2^ > 2σ(*F*^2^)] = 0.041	H-atom parameters constrained
*wR*(*F*^2^) = 0.094	*w* = 1/[σ^2^(*F*_o_^2^) + (0.0372*P*)^2^ + 0.4341*P*] where *P* = (*F*_o_^2^ + 2*F*_c_^2^)/3
*S* = 1.07	(Δ/σ)_max_ < 0.001
3311 reflections	Δρ_max_ = 0.24 e Å^−^^3^
190 parameters	Δρ_min_ = −0.24 e Å^−^^3^
Primary atom site location: structure-invariant direct methods	Extinction correction: none

### Special details

**Table:**

Geometry. All e.s.d.'s (except the e.s.d. in the dihedral angle between two l.s. planes) are estimated using the full covariance matrix. The cell e.s.d.'s are taken into account individually in the estimation of e.s.d.'s in distances, angles and torsion angles; correlations between e.s.d.'s in cell parameters are only used when they are defined by crystal symmetry. An approximate (isotropic) treatment of cell e.s.d.'s is used for estimating e.s.d.'s involving l.s. planes.
Refinement. Refinement of *F*^2^ against ALL reflections. The weighted *R*-factor *wR* and goodness of fit *S* are based on *F*^2^, conventional *R*-factors *R* are based on *F*, with *F* set to zero for negative *F*^2^. The threshold expression of *F*^2^ > 2σ(*F*^2^) is used only for calculating *R*-factors(gt) *etc*. and is not relevant to the choice of reflections for refinement. *R*-factors based on *F*^2^ are statistically about twice as large as those based on *F*, and *R*- factors based on ALL data will be even larger.

### Fractional atomic coordinates and isotropic or equivalent isotropic displacement parameters (Å^2^)

**Table:**

	*x*	*y*	*z*	*U*_iso_*/*U*_eq_	
Fe1	0.19959 (3)	0.79543 (3)	0.08768 (3)	0.03864 (12)	
C10	0.1744 (2)	0.6098 (2)	0.0568 (2)	0.0452 (5)	
H10A	0.1703	0.5456	0.1108	0.054*	
C9	0.2868 (2)	0.6523 (2)	0.03368 (18)	0.0400 (5)	
C15	0.6436 (2)	0.5798 (2)	0.15481 (19)	0.0422 (5)	
C17	0.5244 (2)	0.6339 (2)	0.08206 (18)	0.0404 (5)	
C16	0.4110 (2)	0.6083 (2)	0.09516 (19)	0.0419 (5)	
H16A	0.4124	0.5539	0.1526	0.050*	
N2	0.63206 (19)	0.5064 (2)	0.23633 (19)	0.0559 (6)	
C18	0.5387 (2)	0.7169 (3)	−0.0022 (2)	0.0523 (6)	
C8	0.2495 (2)	0.7467 (2)	−0.04956 (19)	0.0444 (5)	
H8A	0.3056	0.7937	−0.0820	0.053*	
C7	0.1167 (2)	0.7601 (3)	−0.0764 (2)	0.0489 (6)	
H7A	0.0656	0.8192	−0.1301	0.059*	
N1	0.5562 (3)	0.7837 (3)	−0.0659 (2)	0.0831 (9)	
C14	0.7600 (2)	0.6058 (3)	0.1397 (2)	0.0545 (6)	
H1A	0.7658	0.6568	0.0820	0.065*	
C5	0.3262 (2)	0.8697 (3)	0.2228 (2)	0.0551 (7)	
H5A	0.4138	0.8427	0.2532	0.066*	
C11	0.8554 (3)	0.4798 (3)	0.2947 (3)	0.0659 (8)	
H11A	0.9260	0.4443	0.3441	0.079*	
C13	0.8671 (2)	0.5547 (3)	0.2116 (3)	0.0658 (8)	
H13A	0.9460	0.5714	0.2032	0.079*	
C4	0.2841 (3)	0.9625 (2)	0.1432 (2)	0.0570 (7)	
H4A	0.3362	1.0123	0.1086	0.068*	
C6	0.0708 (2)	0.6760 (2)	−0.0113 (2)	0.0494 (6)	
H6A	−0.0172	0.6664	−0.0122	0.059*	
C2	0.1158 (3)	0.8840 (4)	0.1894 (4)	0.0946 (13)	
H2A	0.0298	0.8700	0.1927	0.114*	
C12	0.7372 (3)	0.4577 (3)	0.3038 (3)	0.0667 (8)	
H12A	0.7298	0.4058	0.3604	0.080*	
C3	0.2238 (4)	0.8212 (3)	0.2518 (2)	0.0751 (10)	
H3A	0.2268	0.7556	0.3063	0.090*	
C1	0.1528 (3)	0.9719 (3)	0.1227 (3)	0.0840 (11)	
H1B	0.0969	1.0294	0.0707	0.101*	

### Atomic displacement parameters (Å^2^)

**Table:**

	*U*^11^	*U*^22^	*U*^33^	*U*^12^	*U*^13^	*U*^23^
Fe1	0.03100 (18)	0.0435 (2)	0.0417 (2)	−0.00034 (14)	0.01115 (13)	−0.00617 (15)
C10	0.0451 (13)	0.0435 (13)	0.0463 (13)	−0.0074 (10)	0.0122 (10)	−0.0034 (11)
C9	0.0417 (12)	0.0401 (12)	0.0412 (12)	−0.0010 (10)	0.0167 (10)	−0.0059 (10)
C15	0.0434 (13)	0.0364 (12)	0.0505 (14)	0.0016 (10)	0.0195 (11)	−0.0089 (10)
C17	0.0461 (13)	0.0351 (12)	0.0445 (13)	0.0016 (10)	0.0204 (10)	−0.0062 (10)
C16	0.0459 (13)	0.0381 (12)	0.0454 (13)	0.0026 (10)	0.0193 (10)	−0.0023 (10)
N2	0.0445 (12)	0.0576 (13)	0.0678 (14)	0.0062 (10)	0.0198 (11)	0.0123 (11)
C18	0.0431 (14)	0.0598 (17)	0.0593 (16)	0.0034 (12)	0.0233 (12)	0.0040 (13)
C8	0.0453 (13)	0.0511 (13)	0.0397 (12)	0.0000 (11)	0.0170 (11)	−0.0011 (11)
C7	0.0461 (14)	0.0572 (15)	0.0388 (13)	0.0002 (11)	0.0051 (11)	−0.0016 (11)
N1	0.0676 (17)	0.100 (2)	0.090 (2)	0.0035 (15)	0.0367 (15)	0.0364 (17)
C14	0.0470 (14)	0.0577 (16)	0.0645 (17)	0.0010 (12)	0.0255 (13)	−0.0014 (13)
C5	0.0505 (14)	0.0611 (17)	0.0459 (14)	0.0000 (13)	0.0016 (12)	−0.0139 (13)
C11	0.0457 (15)	0.0678 (19)	0.081 (2)	0.0107 (14)	0.0126 (14)	0.0006 (16)
C13	0.0404 (14)	0.075 (2)	0.085 (2)	0.0026 (14)	0.0225 (14)	−0.0074 (17)
C4	0.0566 (16)	0.0460 (15)	0.0618 (17)	−0.0087 (12)	0.0072 (13)	−0.0111 (13)
C6	0.0359 (12)	0.0571 (16)	0.0512 (14)	−0.0097 (11)	0.0066 (11)	−0.0082 (12)
C2	0.058 (2)	0.114 (3)	0.126 (3)	−0.019 (2)	0.050 (2)	−0.073 (3)
C12	0.0573 (17)	0.068 (2)	0.0741 (19)	0.0057 (14)	0.0184 (15)	0.0180 (15)
C3	0.107 (3)	0.079 (2)	0.0500 (17)	−0.017 (2)	0.0393 (18)	−0.0222 (15)
C1	0.064 (2)	0.061 (2)	0.106 (3)	0.0237 (16)	−0.0094 (19)	−0.0369 (19)

### Geometric parameters (Å, °)

**Table:**

Fe1—C10	2.031 (2)	C8—C7	1.421 (3)
Fe1—C2	2.032 (3)	C8—H8A	0.9800
Fe1—C5	2.034 (2)	C7—C6	1.414 (4)
Fe1—C9	2.036 (2)	C7—H7A	0.9800
Fe1—C3	2.037 (3)	C14—C13	1.383 (4)
Fe1—C8	2.042 (2)	C14—H1A	0.9300
Fe1—C4	2.048 (3)	C5—C3	1.393 (4)
Fe1—C7	2.050 (2)	C5—C4	1.397 (4)
Fe1—C6	2.052 (2)	C5—H5A	0.9800
Fe1—C1	2.044 (3)	C11—C13	1.361 (4)
C10—C6	1.411 (3)	C11—C12	1.372 (4)
C10—C9	1.438 (3)	C11—H11A	0.9300
C10—H10A	0.9800	C13—H13A	0.9300
C9—C8	1.432 (3)	C4—C1	1.408 (4)
C9—C16	1.450 (3)	C4—H4A	0.9800
C15—N2	1.334 (3)	C6—H6A	0.9800
C15—C14	1.389 (3)	C2—C1	1.404 (5)
C15—C17	1.492 (3)	C2—C3	1.401 (5)
C17—C16	1.346 (3)	C2—H2A	0.9800
C17—C18	1.433 (3)	C12—H12A	0.9300
C16—H16A	0.9300	C3—H3A	0.9800
N2—C12	1.336 (3)	C1—H1B	0.9800
C18—N1	1.138 (3)		
			
C10—Fe1—C2	121.13 (14)	C17—C16—H16A	114.9
C10—Fe1—C5	124.61 (11)	C9—C16—H16A	114.9
C2—Fe1—C5	67.36 (12)	C15—N2—C12	117.6 (2)
C10—Fe1—C9	41.40 (9)	N1—C18—C17	176.5 (3)
C2—Fe1—C9	156.35 (17)	C7—C8—C9	107.8 (2)
C5—Fe1—C9	107.32 (10)	C7—C8—Fe1	69.98 (14)
C10—Fe1—C3	107.43 (12)	C9—C8—Fe1	69.22 (13)
C2—Fe1—C3	40.27 (14)	C7—C8—H8A	126.1
C5—Fe1—C3	40.03 (12)	C9—C8—H8A	126.1
C9—Fe1—C3	120.76 (13)	Fe1—C8—H8A	126.1
C10—Fe1—C8	68.99 (10)	C6—C7—C8	108.7 (2)
C2—Fe1—C8	161.54 (16)	C6—C7—Fe1	69.92 (14)
C5—Fe1—C8	121.64 (11)	C8—C7—Fe1	69.38 (14)
C9—Fe1—C8	41.13 (9)	C6—C7—H7A	125.6
C3—Fe1—C8	156.49 (13)	C8—C7—H7A	125.6
C10—Fe1—C4	161.07 (10)	Fe1—C7—H7A	125.6
C2—Fe1—C4	67.80 (14)	C13—C14—C15	119.1 (3)
C5—Fe1—C4	40.01 (11)	C13—C14—H1A	120.5
C9—Fe1—C4	123.96 (10)	C15—C14—H1A	120.5
C3—Fe1—C4	67.64 (12)	C3—C5—C4	109.2 (3)
C8—Fe1—C4	107.79 (11)	C3—C5—Fe1	70.08 (16)
C10—Fe1—C7	68.15 (10)	C4—C5—Fe1	70.54 (14)
C2—Fe1—C7	125.25 (14)	C3—C5—H5A	125.4
C5—Fe1—C7	157.24 (11)	C4—C5—H5A	125.4
C9—Fe1—C7	68.70 (10)	Fe1—C5—H5A	125.4
C3—Fe1—C7	161.43 (13)	C13—C11—C12	118.5 (3)
C8—Fe1—C7	40.64 (9)	C13—C11—H11A	120.8
C4—Fe1—C7	122.35 (11)	C12—C11—H11A	120.8
C10—Fe1—C6	40.43 (9)	C11—C13—C14	119.1 (3)
C2—Fe1—C6	108.25 (12)	C11—C13—H13A	120.5
C5—Fe1—C6	161.08 (11)	C14—C13—H13A	120.5
C9—Fe1—C6	68.88 (10)	C5—C4—C1	107.2 (3)
C3—Fe1—C6	124.75 (12)	C5—C4—Fe1	69.45 (15)
C8—Fe1—C6	68.49 (10)	C1—C4—Fe1	69.68 (16)
C4—Fe1—C6	157.37 (11)	C5—C4—H4A	126.4
C7—Fe1—C6	40.34 (10)	C1—C4—H4A	126.4
C10—Fe1—C1	156.70 (13)	Fe1—C4—H4A	126.4
C2—Fe1—C1	40.31 (16)	C10—C6—C7	108.0 (2)
C5—Fe1—C1	67.21 (12)	C10—C6—Fe1	68.98 (13)
C9—Fe1—C1	161.07 (15)	C7—C6—Fe1	69.74 (14)
C3—Fe1—C1	67.60 (15)	C10—C6—H6A	126.0
C8—Fe1—C1	124.84 (15)	C7—C6—H6A	126.0
C4—Fe1—C1	40.26 (12)	Fe1—C6—H6A	126.0
C7—Fe1—C1	108.88 (13)	C1—C2—C3	108.0 (3)
C6—Fe1—C1	122.26 (12)	C1—C2—Fe1	70.29 (18)
C6—C10—C9	108.5 (2)	C3—C2—Fe1	70.05 (17)
C6—C10—Fe1	70.59 (14)	C1—C2—H2A	126.0
C9—C10—Fe1	69.48 (13)	C3—C2—H2A	126.0
C6—C10—H10A	125.7	Fe1—C2—H2A	126.0
C9—C10—H10A	125.7	N2—C12—C11	123.9 (3)
Fe1—C10—H10A	125.7	N2—C12—H12A	118.0
C8—C9—C10	107.0 (2)	C11—C12—H12A	118.0
C8—C9—C16	130.6 (2)	C5—C3—C2	107.6 (3)
C10—C9—C16	122.2 (2)	C5—C3—Fe1	69.89 (16)
C8—C9—Fe1	69.65 (13)	C2—C3—Fe1	69.68 (19)
C10—C9—Fe1	69.11 (13)	C5—C3—H3A	126.2
C16—C9—Fe1	122.35 (16)	C2—C3—H3A	126.2
N2—C15—C14	121.9 (2)	Fe1—C3—H3A	126.2
N2—C15—C17	116.1 (2)	C2—C1—C4	108.0 (3)
C14—C15—C17	122.0 (2)	C2—C1—Fe1	69.40 (18)
C16—C17—C18	122.0 (2)	C4—C1—Fe1	70.06 (15)
C16—C17—C15	122.5 (2)	C2—C1—H1B	126.0
C18—C17—C15	115.5 (2)	C4—C1—H1B	126.0
C17—C16—C9	130.1 (2)	Fe1—C1—H1B	126.0
			
C2—Fe1—C10—C6	81.7 (2)	C3—Fe1—C5—C4	−119.9 (3)
C5—Fe1—C10—C6	164.22 (16)	C8—Fe1—C5—C4	79.87 (19)
C9—Fe1—C10—C6	−119.4 (2)	C7—Fe1—C5—C4	46.5 (4)
C3—Fe1—C10—C6	123.55 (18)	C6—Fe1—C5—C4	−162.1 (3)
C8—Fe1—C10—C6	−81.09 (16)	C1—Fe1—C5—C4	−38.1 (2)
C4—Fe1—C10—C6	−164.7 (3)	C12—C11—C13—C14	0.0 (4)
C7—Fe1—C10—C6	−37.29 (15)	C15—C14—C13—C11	0.4 (4)
C1—Fe1—C10—C6	49.9 (4)	C3—C5—C4—C1	0.1 (3)
C2—Fe1—C10—C9	−158.91 (18)	Fe1—C5—C4—C1	59.75 (18)
C5—Fe1—C10—C9	−76.36 (17)	C3—C5—C4—Fe1	−59.64 (19)
C3—Fe1—C10—C9	−117.04 (17)	C10—Fe1—C4—C5	−41.3 (4)
C8—Fe1—C10—C9	38.33 (13)	C2—Fe1—C4—C5	80.8 (2)
C4—Fe1—C10—C9	−45.3 (4)	C9—Fe1—C4—C5	−75.9 (2)
C7—Fe1—C10—C9	82.13 (15)	C3—Fe1—C4—C5	37.08 (18)
C6—Fe1—C10—C9	119.4 (2)	C8—Fe1—C4—C5	−118.34 (17)
C1—Fe1—C10—C9	169.3 (3)	C7—Fe1—C4—C5	−160.60 (16)
C6—C10—C9—C8	0.4 (3)	C6—Fe1—C4—C5	165.0 (3)
Fe1—C10—C9—C8	−59.63 (16)	C1—Fe1—C4—C5	118.3 (3)
C6—C10—C9—C16	175.9 (2)	C10—Fe1—C4—C1	−159.7 (3)
Fe1—C10—C9—C16	115.9 (2)	C2—Fe1—C4—C1	−37.6 (2)
C6—C10—C9—Fe1	60.04 (17)	C5—Fe1—C4—C1	−118.3 (3)
C10—Fe1—C9—C8	118.34 (19)	C9—Fe1—C4—C1	165.8 (2)
C2—Fe1—C9—C8	168.5 (3)	C3—Fe1—C4—C1	−81.3 (2)
C5—Fe1—C9—C8	−118.57 (15)	C8—Fe1—C4—C1	123.3 (2)
C3—Fe1—C9—C8	−160.17 (16)	C7—Fe1—C4—C1	81.1 (2)
C4—Fe1—C9—C8	−77.80 (17)	C6—Fe1—C4—C1	46.7 (4)
C7—Fe1—C9—C8	37.66 (14)	C9—C10—C6—C7	−0.4 (3)
C6—Fe1—C9—C8	81.07 (15)	Fe1—C10—C6—C7	58.99 (17)
C1—Fe1—C9—C8	−48.5 (4)	C9—C10—C6—Fe1	−59.35 (16)
C2—Fe1—C9—C10	50.1 (3)	C8—C7—C6—C10	0.2 (3)
C5—Fe1—C9—C10	123.08 (15)	Fe1—C7—C6—C10	−58.51 (17)
C3—Fe1—C9—C10	81.49 (18)	C8—C7—C6—Fe1	58.70 (17)
C8—Fe1—C9—C10	−118.34 (19)	C2—Fe1—C6—C10	−116.9 (2)
C4—Fe1—C9—C10	163.86 (15)	C5—Fe1—C6—C10	−43.6 (4)
C7—Fe1—C9—C10	−80.68 (15)	C9—Fe1—C6—C10	38.14 (14)
C6—Fe1—C9—C10	−37.27 (14)	C3—Fe1—C6—C10	−75.4 (2)
C1—Fe1—C9—C10	−166.9 (3)	C8—Fe1—C6—C10	82.44 (15)
C10—Fe1—C9—C16	−115.7 (2)	C4—Fe1—C6—C10	167.2 (3)
C2—Fe1—C9—C16	−65.6 (4)	C7—Fe1—C6—C10	119.7 (2)
C5—Fe1—C9—C16	7.4 (2)	C1—Fe1—C6—C10	−159.0 (2)
C3—Fe1—C9—C16	−34.2 (2)	C10—Fe1—C6—C7	−119.7 (2)
C8—Fe1—C9—C16	125.9 (3)	C2—Fe1—C6—C7	123.4 (2)
C4—Fe1—C9—C16	48.1 (2)	C5—Fe1—C6—C7	−163.3 (3)
C7—Fe1—C9—C16	163.6 (2)	C9—Fe1—C6—C7	−81.56 (16)
C6—Fe1—C9—C16	−153.0 (2)	C3—Fe1—C6—C7	164.90 (18)
C1—Fe1—C9—C16	77.4 (4)	C8—Fe1—C6—C7	−37.26 (15)
N2—C15—C17—C16	−1.7 (3)	C4—Fe1—C6—C7	47.5 (4)
C14—C15—C17—C16	179.2 (2)	C1—Fe1—C6—C7	81.3 (2)
N2—C15—C17—C18	177.4 (2)	C10—Fe1—C2—C1	−161.20 (17)
C14—C15—C17—C18	−1.6 (3)	C5—Fe1—C2—C1	81.0 (2)
C18—C17—C16—C9	0.5 (4)	C9—Fe1—C2—C1	162.4 (2)
C15—C17—C16—C9	179.6 (2)	C3—Fe1—C2—C1	118.7 (3)
C8—C9—C16—C17	−8.6 (4)	C8—Fe1—C2—C1	−42.1 (5)
C10—C9—C16—C17	177.1 (2)	C4—Fe1—C2—C1	37.53 (18)
Fe1—C9—C16—C17	−98.6 (3)	C7—Fe1—C2—C1	−77.3 (2)
C14—C15—N2—C12	−0.1 (4)	C6—Fe1—C2—C1	−118.69 (19)
C17—C15—N2—C12	−179.2 (2)	C10—Fe1—C2—C3	80.1 (2)
C10—C9—C8—C7	−0.3 (3)	C5—Fe1—C2—C3	−37.71 (19)
C16—C9—C8—C7	−175.3 (2)	C9—Fe1—C2—C3	43.7 (4)
Fe1—C9—C8—C7	−59.58 (17)	C8—Fe1—C2—C3	−160.8 (3)
C10—C9—C8—Fe1	59.29 (15)	C4—Fe1—C2—C3	−81.2 (2)
C16—C9—C8—Fe1	−115.7 (2)	C7—Fe1—C2—C3	164.06 (18)
C10—Fe1—C8—C7	80.51 (16)	C6—Fe1—C2—C3	122.6 (2)
C2—Fe1—C8—C7	−46.3 (4)	C1—Fe1—C2—C3	−118.7 (3)
C5—Fe1—C8—C7	−160.94 (16)	C15—N2—C12—C11	0.6 (5)
C9—Fe1—C8—C7	119.1 (2)	C13—C11—C12—N2	−0.6 (5)
C3—Fe1—C8—C7	166.0 (3)	C4—C5—C3—C2	0.2 (3)
C4—Fe1—C8—C7	−119.28 (16)	Fe1—C5—C3—C2	−59.7 (2)
C6—Fe1—C8—C7	36.99 (15)	C4—C5—C3—Fe1	59.92 (18)
C1—Fe1—C8—C7	−78.15 (19)	C1—C2—C3—C5	−0.4 (3)
C10—Fe1—C8—C9	−38.57 (13)	Fe1—C2—C3—C5	59.86 (19)
C2—Fe1—C8—C9	−165.4 (3)	C1—C2—C3—Fe1	−60.3 (2)
C5—Fe1—C8—C9	79.98 (17)	C10—Fe1—C3—C5	123.48 (17)
C3—Fe1—C8—C9	46.9 (3)	C2—Fe1—C3—C5	−118.6 (3)
C4—Fe1—C8—C9	121.64 (15)	C9—Fe1—C3—C5	80.2 (2)
C7—Fe1—C8—C9	−119.1 (2)	C8—Fe1—C3—C5	46.2 (4)
C6—Fe1—C8—C9	−82.09 (15)	C4—Fe1—C3—C5	−37.06 (17)
C1—Fe1—C8—C9	162.77 (15)	C7—Fe1—C3—C5	−163.4 (3)
C9—C8—C7—C6	0.1 (3)	C6—Fe1—C3—C5	164.61 (16)
Fe1—C8—C7—C6	−59.03 (18)	C1—Fe1—C3—C5	−80.8 (2)
C9—C8—C7—Fe1	59.10 (16)	C10—Fe1—C3—C2	−117.9 (2)
C10—Fe1—C7—C6	37.37 (15)	C5—Fe1—C3—C2	118.6 (3)
C2—Fe1—C7—C6	−76.1 (2)	C9—Fe1—C3—C2	−161.2 (2)
C5—Fe1—C7—C6	166.1 (3)	C8—Fe1—C3—C2	164.8 (3)
C9—Fe1—C7—C6	82.05 (16)	C4—Fe1—C3—C2	81.6 (2)
C3—Fe1—C7—C6	−42.2 (4)	C7—Fe1—C3—C2	−44.8 (5)
C8—Fe1—C7—C6	120.1 (2)	C6—Fe1—C3—C2	−76.8 (2)
C4—Fe1—C7—C6	−160.39 (15)	C1—Fe1—C3—C2	37.9 (2)
C1—Fe1—C7—C6	−117.96 (19)	C3—C2—C1—C4	0.5 (3)
C10—Fe1—C7—C8	−82.78 (16)	Fe1—C2—C1—C4	−59.6 (2)
C2—Fe1—C7—C8	163.7 (2)	C3—C2—C1—Fe1	60.1 (2)
C5—Fe1—C7—C8	46.0 (3)	C5—C4—C1—C2	−0.4 (3)
C9—Fe1—C7—C8	−38.10 (15)	Fe1—C4—C1—C2	59.2 (2)
C3—Fe1—C7—C8	−162.4 (4)	C5—C4—C1—Fe1	−59.61 (19)
C4—Fe1—C7—C8	79.47 (18)	C10—Fe1—C1—C2	44.2 (4)
C6—Fe1—C7—C8	−120.1 (2)	C5—Fe1—C1—C2	−81.4 (2)
C1—Fe1—C7—C8	121.90 (19)	C9—Fe1—C1—C2	−158.1 (3)
N2—C15—C14—C13	−0.4 (4)	C3—Fe1—C1—C2	−37.8 (2)
C17—C15—C14—C13	178.6 (2)	C8—Fe1—C1—C2	165.02 (19)
C10—Fe1—C5—C3	−75.2 (2)	C4—Fe1—C1—C2	−119.2 (3)
C2—Fe1—C5—C3	37.9 (2)	C7—Fe1—C1—C2	122.7 (2)
C9—Fe1—C5—C3	−117.5 (2)	C6—Fe1—C1—C2	80.1 (2)
C8—Fe1—C5—C3	−160.23 (19)	C10—Fe1—C1—C4	163.4 (3)
C4—Fe1—C5—C3	119.9 (3)	C2—Fe1—C1—C4	119.2 (3)
C7—Fe1—C5—C3	166.4 (3)	C5—Fe1—C1—C4	37.87 (18)
C6—Fe1—C5—C3	−42.2 (4)	C9—Fe1—C1—C4	−38.8 (5)
C1—Fe1—C5—C3	81.8 (2)	C3—Fe1—C1—C4	81.4 (2)
C10—Fe1—C5—C4	164.91 (16)	C8—Fe1—C1—C4	−75.8 (2)
C2—Fe1—C5—C4	−82.0 (2)	C7—Fe1—C1—C4	−118.11 (19)
C9—Fe1—C5—C4	122.60 (17)	C6—Fe1—C1—C4	−160.66 (17)

### Hydrogen-bond geometry (Å, °)

**Table:**

*D*—H···*A*	*D*—H	H···*A*	*D*···*A*	*D*—H···*A*
C16—H16A···N2	0.93	2.41	2.804 (3)	105
C4—H4A···N1^i^	0.98	2.62	3.538 (4)	156

Symmetry codes: (i) −*x*+1, −*y*+2, −*z*.
